# Investigating Maternal Perception of Control and Support in the Postpartum Period and Influencing Factors in Childbirth: A Multiple Linear Regression Analysis Model

**DOI:** 10.1002/nop2.70095

**Published:** 2025-01-07

**Authors:** Tuğba Yazici Topçu, Ruveyde Aydin, Songül Aktaş

**Affiliations:** ^1^ Farabi Hospital, Newborn Intensive Care Unit Karadeniz Technical University Trabzon Türkiye; ^2^ Faculty of Health Sciences, Maternity and Gynaecological Nursing Department Ondokuz Mayıs University Samsun Türkiye; ^3^ Faculty of Health Sciences, Department of Midwifery Karadeniz Technical University Trabzon Türkiye

**Keywords:** birth, birth satisfaction, control, midwife, mother, postpartum, support

## Abstract

**Aim:**

This study aimed to assess the levels of maternal perception of control and support during birth and the factors influencing them in the postpartum period.

**Design:**

A cross‐sectional design was employed. The STROBE checklist was used.

**Methods:**

The study was conducted between July 2021 and March 2022 with 400 mothers who were in their first 2 months postpartum and lived in Türkiye. Data were collected online using a Maternal Descriptive Information Form and the Support and Control in Birth (SCIB) Questionnaire. Descriptive statistics, such as percentage, mean, standard deviation and multiple linear regression analysis, were employed for data evaluation.

**Results:**

The mean score on the total SCIB was 92.4 ± 13.01. Vaginal birth and caesarean section accounted for 47.3% and 52.7% of the study group, respectively, with only 35% being assisted by a midwife. Variables of age, education level, number of pregnancies, mode of birth and health professionals assisting birth showed no significant effect on the total SCIB score (*p* > 0.05). Maternal satisfaction with birth scores, satisfaction with health professionals involved birth and fulfilled expectations from health professionals were positively associated with high SCIB scores (*p* < 0.05).

**Conclusion:**

The level of SCIB in our study fell within a ‘moderate’ range, considering the lowest and highest scores on the scale. Notably, increased satisfaction with birth experience and healthcare professionals, positively influenced maternal perceptions of control and support during birth. In contrast, certain socio‐demographic and obstetric characteristics did not demonstrate a significant impact. To enhance the sense of support and control during childbirth, it is crucial to identify and fulfil the expectations of mothers from midwives and birth supportive care to ultimately elevate maternal satisfaction in the childbirth experience.

**Patient or Public Contribution:**

None.

## 1 Introduction

During the birth process, women endeavour to adapt to the birth environment and manage the associated pains. Effective birth control and decision making in birth practices and care may necessitate the support of health professionals as well as family and relatives (Bakhteh et al. 2023; Shahveisi, Nourizadeh, and Mehrabi 2023). The level of control and support experienced during birth significantly contributes to maternal satisfaction (Aktaş and Pasinlioğlu 2021), fostering a positive birth experience and protecting and promoting perinatal mental health (Colley et al. 2018; Leinweber et al. 2023). The concept of birth control is commonly linked to a woman's capacity to cope with birth pain and her active role in the birth process. Two types of control, external and internal, are recognised in this context (Karlström, Nysted, and Hildingsson 2015). External control involves a woman's perception of losing control over external factors, such as analgesia, information, environment, decisions, procedures and the birth process itself. Internal control encompasses a woman's ability to exert influence over her experiences, feelings, thoughts and physical functionality (Ford, Ayers, and Wright 2009; ‐ Inci, GokceIsbir, and Tanhan 2015; Cevik et al. 2023).

## 2 Background

The quality of intrapartum care plays a crucial role in shaping maternal perceptions of control and support during birth. Enhancement of this perception has several positive outcomes, including an increased ability to cope with birth pain, decreased fear of birth, reduced need for medical interventions (Çankaya and Can 2021), preservation of the physiological aspects of birth, increased satisfaction with birth (Zielinski, Brody, and Low 2016; Aktaş and Küçük Alemdar 2024), improved maternal adaptation and a positive transition to parenthood (Olza et al. 2020; Buback et al. 2022; Demirel, Kaya, and Evcili 2022). A study conducted with 725 mothers in Türkiye showed that the high level of perception of support and control at birth decreased the fear of birth and increased the satisfaction levels of puerperal women in vaginal and caesarean births (Demirel, Kaya, and Evcili 2022). Various factors, such as socio‐demographic and obstetric characteristics (Bakhteh et al. 2023; Demirel, Kaya, and Evcili 2022; Aynaci 2020; Colley et al. 2018), perception of birth, interventions applied during birth, birth environment, presence of social support, cultural characteristics (Bohren et al. 2019; Striebich and Ayerle 2020; Buback et al. 2022; Demir 2023), quality of antenatal and intrapartum care, attitudes and behaviour of the midwife and obstetrician assisting the birth (Çankaya and Can 2021; Aktaş and Küçük Alemdar 2024) and natural disasters (such as migration, pandemic), can affect maternal perception of control and support during birth (Mortazavi and Mehrabadi 2024; Toker and Aktaş 2021). In a scoping review on the examination of 13 studies, it was stated that the birth environment where women felt like they were at home provided them with physical and psychological support and increased their perception of control during birth (Chen et al. 2023). In a Cochrane systematic review including 52 studies, it was revealed that the compassionate approach to mothers by people accompanying the birth provided them with emotional support, increased their sense of control and contributed to a positive birth experience (Bohren et al. 2019).

A low level of perception of support and control during birth can lead to negative outcomes, such as inability to cope with labour pain, increased need for medical interventions and requests for caesarean section and negative birth experiences (Cevik et al. 2023; Striebich and Ayerle 2020; Inci, GokceIsbir, and Tanhan 2015). In a study conducted with 2541 women in Sweden, it was determined that women's lack of sense of control during birth and inadequate support from partners and healthcare professionals were the most important risk factors for a negative birth experience (Waldenström et al. 2004). Negative birth experiences have been linked to posttraumatic stress disorder, challenges in breastfeeding and mother–baby attachment, traumatic perceptions of birth and increased fears related to subsequent births (Aktaş and Aydın 2019; Viirman et al. 2023).

Perceived support plays a pivotal role in enhancing women's sense of control during birth, contributing to positive birth experiences. The primary sources of support during birth include support from spouses or partners, the person accompanying the birth and health professionals (Buback et al. 2022; Akgün and Aktaş 2023). The support system encompasses various elements, including coping techniques, personnel attitudes, empathy, understanding, assurance, encouragement, listening, informative support and overall perception of support (Ford and Ayers 2011; Demirel, Kaya, and Evcili 2022; Cevik et al. 2023). Mothers have expectations from midwives and obstetricians like physical and psychosocial support (communication, pain relief and respectful care), and meeting these expectations not only contributes to a positive birth experience but also enhances the overall sense of control and support (Aktaş and Pasinlioğlu 2017; Turan, Suveren, and Vural 2022; Bakhteh et al. 2023). In a qualitative study, some mothers whose expectations for physical and emotional support from midwives during vaginal birth were met stated that this support helped them to cope with labour pain better, increased their belief in successful birth and birth control and augmented their satisfaction with the midwife and birth and that they recommended vaginal birth to others (Aktaş and Küçük Alemdar 2024).

The WHO (2018) recommends improving the quality of intrapartum care to promote positive birth experiences. The concept of a positive birth experience comprises crucial components, such as woman‐centred care and feeling supported, safe, in control and respected (Leinweber et al. 2023). A positive birth experience not only improves women's health during the perinatal period, but also extends its positive impact beyond promoting health, both during and following the perinatal period (Hildingsson et al. 2013; Leinweber et al. 2023).

This study was conducted to determine the levels of the perception of control and support at birth and to examine the factors affecting these levels. There was no research in the literature on the investigation of the perceived levels of control and support during birth and various factors affecting them among low‐risk mothers in the first 2 months postpartum. Therefore, this study is anticipated to address this gap in the existing literature and provide insights for future research. Knowing the levels of maternal perception of support and control during birth and the factors affecting them contributes to increasing the quality of midwifery care and may be helpful for better planning to improve the quality of birth centres. Moreover, the outcomes of this study are anticipated to contribute to the existing body of literature, furnish valuable insights for subsequent research and align with the strategies outlined by the World Health Organisation (WHO) in 2018. These strategies aim to enhance the quality of intrapartum midwifery care, foster positive birth experiences and promote improved perinatal mental health.

### 2.1 Research Questions

1. What are the levels of maternal perceptions of support and control during birth?
2. What are the factors influencing the levels of maternal perceptions of support and control during birth?

## 3 Methods

### 3.1 Design

A cross‐sectional design was employed. The study data were collected online and the Strengthening the Reporting of Observational Studies in Epidemiology (STROBE) guidelines were followed (Vandenbroucke et al. 2007) (see Supporting Information S1).

### 3.2 Sample/Participants

The population of the study consisted of mothers who lived in Türkiye and were in their first 2 months postpartum. Mothers living in seven different regions of Türkiye were included in the study (there are seven regions in Türkiye in total).

The inclusion criteria were volunteering to participate in the study, having a minimum of primary school education, being 20 years of age or older, having proficiency in Turkish, having a mobile phone with the WhatsApp application, giving birth between the 38th to 41st weeks of gestation, being in the first 2 months (8 weeks) of the postpartum period, having been identified as low risk during birth (single pregnancy, absence of systemic diseases such as severe preeclampsia or gestational diabetes), having no diagnosed psychiatric disease during the birth and postpartum period and having a healthy newborn.

**FIGURE 1:**
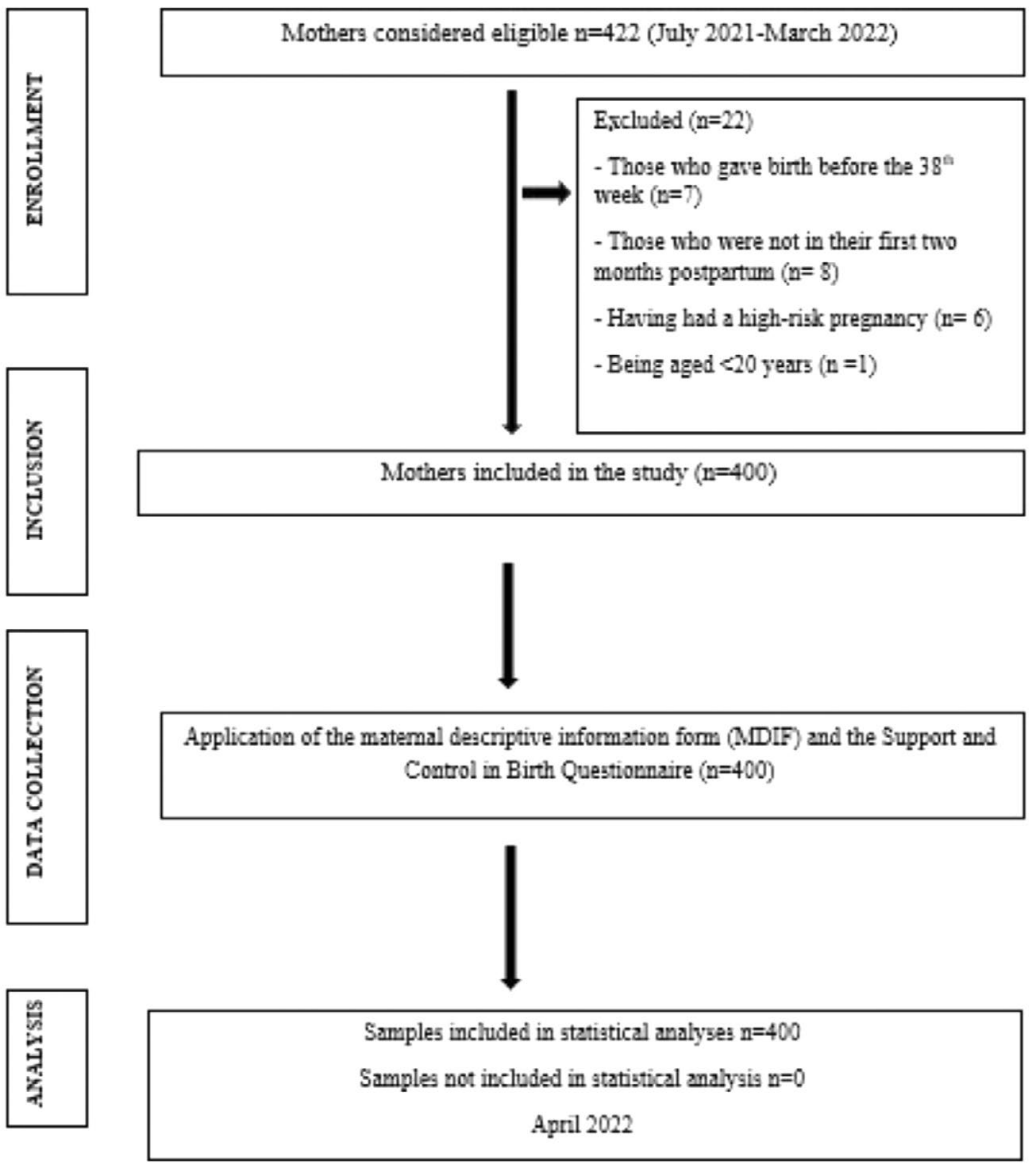
Flow chart of the study.

The exclusion criteria were giving birth before the 37th or after the ≥ 42nd weeks of gestation or being a migrant mother. Twenty‐two mothers were excluded from the study because of various reasons. Figure 1 shows information such as the number of mothers included and excluded from the study, the reasons for the mothers not included, etc. The flow chart of the study is shown in Figure 1.

The sample size of the study was determined on the OpenEpi Version 3.01 software by calculating proportion. In this study, the sample size was calculated as 384 subjects using the sampling of the unknown population formula, based on a prevalence of 50% and a confidence interval of 95%. Considering some attrition, approximately 4% more subjects than the determined sample size was recruited and a total of 400 mothers constituted the sample of the study.

### 3.3 Data Collection

Initially, all researchers shared the research invitation link on their social media accounts, such as ‘Facebook, X, Instagram and WhatsApp’. This invitation outlined key details, such as the name, purpose, sampling criteria and data collection methods of the study, and the researcher's contact information. To reach the sample in the study, the researchers sent a request to online groups about ‘pregnancy, motherhood and breastfeeding’ that were accessible on social media to join them, in addition to sharing the research invitation link on their personal social media accounts. They shared the link to the online survey in the groups that responded positively to this request. In this study, data were collected from mothers living in seven different regions of Türkiye. The descriptive information form included a question about the region where the mothers lived. One of the purposes of conducting an online survey in the study was to examine the support and control levels and factors influencing them during birth for mothers from all regions of Türkiye.

### 3.4 Instrument

Study data were collected between July 2021 and March 2022 with an online questionnaire created on Google Forms. The first page of the Google form included information about the research (such as the purpose of the study, sample group and data collection methods) and a voluntary consent form to participate in the study. The data of the mothers who selected the option ‘I agree to participate in the study’ were collected via this online questionnaire involving three forms: a Demographic Information Form, a Maternal Descriptive Information Form (MDIF) and the Support and Control in Birth Questionnaire (SCIB) respectively.

#### 3.4.1 The Demographic Information Form

This form consisted of questions about mothers that would be used for the inclusion criteria of the study. They were yes–no type questions, such as ‘Are you 20 years old or older? Are you an immigrant? Do you speak and understand Turkish? Did you give birth between the 38th and 41st weeks of gestation? Do you have a mobile phone with a WhatsApp application? Did you have a high‐risk birth (presence of problems such as joint disease, heart disease or multiple pregnancies)? Is your baby healthy now?’ Mothers who responded ‘yes’ to these questions were able to proceed to the Maternal Descriptive Information Form. Those who responded ‘no’ to the items were included in the group of mothers who did not meet the sampling criteria.

#### 3.4.2 The Maternal Descriptive Information Form (MDIF)

This form prepared by researchers in line with the literature, consists of 16 questions about mothers' socio‐demographic and obstetric characteristics (Inci, GokceIsbir, and Tanhan 2015; Colley et al. 2018; Aynaci 2020), such as age, education, employment status, perceived income (low, medium or high), family type (nuclear family or extended family), place of residence (village, town or city), type of pregnancy (natural or with the help of reproductive techniques), number of births, mode of the current birth (vaginal or caesarean), postpartum week, level of satisfaction with the present birth and the health professional (obstetrician, midwife) who assisted the birth (assessment with a visual analogue scale with a 1 to 10 rating) and the level of fulfilment of expectations by health professionals during birth (yes, no or partially). In our study, mothers who completed the MDIF form moved on to the Support and Control in Birth Questionnaire.

#### 3.4.3 The Support and Control in Birth Questionnaire (SCIB)

The SCIB was originally developed by Ford, Ayers and Wright (2009) and was later adapted into Turkish by Inci, GokceIsbir, and Tanhan (2015). It has 33 five‐point Likert‐type items scored with options from 5 = completely agree to 1 = completely disagree and three sub‐dimensions, namely ‘perception of support’, ‘perception of external control’ and ‘perception of internal control’.

The perception of support sub‐dimension is used to evaluate the woman's communication with her environment and health professionals and the support she receives from them. This sub‐dimension has 17 items, for example, ‘The staff encouraged me to try new ways of coping’ and ‘The staff knew intuitively what I wanted or needed’. The score range of this sub‐dimension is 17 to 84. The perception of internal control sub‐dimension is utilised to assess the woman's ability to control her body during birth and her responses to pain. This sub‐dimension includes 10 items such as ‘I could control my response to pain’ and ‘I was under the influence of negative emotions’. Scores on this sub‐dimension range from 10 to 50. The perception of external control sub‐dimension is employed to examine the woman's participation in decisions, such as medical interventions applied during birth, the application time of these interventions and the individuals who will be present in the birthing room. This sub‐dimension includes six items such as ‘I had control over the decisions to be made’ and ‘I had control over when the procedures would be performed’. Scores on the sub‐dimension vary from 6 to 30.

The total scale score ranges from 33 to 165, with higher scores indicating high levels of support and control at birth. The sub‐dimensions and their items are shown in Table 1. In the internal consistency reliability analysis of the scale, Cronbach's alpha coefficients were found as 0.84 for the perception of support sub‐dimension, 0.83 for the external control sub‐dimension, 0.87 for the internal control sub‐dimension and 0.84 for the total SCIB (*p* < 0.001). The test–retest (rho) coefficient estimated as a result of the analysis conducted to assess the temporal stability of the SCIB was 0.86 for the total scale (*p* < 0.001).

**TABLE 1:** Mean scores on the total and sub‐dimensions of the support and control in birth questionnaire.

Scale	Minimum	Maximum	Mean	SD
The Support and Control in Birth Questionnaire (total)	39	138	92.4	13.01
Sub‐dimensions				
Perception of support	19	78	46.6	9.07
Perception of external control	6	30	18.8	3.43
Perception of internal control	14	41	27.7	4.16
Perception of support items				
1. The staff helped me to find the energy to continue when I wanted to give up	1	5	2.2	1.01
2. The staff knew intuitively what I wanted or needed	1	5	2.5	1.07
3. The staff made efforts to comfort me	1	5	2.4	1.09
4. The staff encouraged me to try new ways of coping	1	5	2.4	1.10
5. The staff encouraged me to not fight against what my body does naturally	1	5	2.4	1.10
6. The staff realised the pain I felt	1	5	2.8	1.30
7. I felt that the staff had their own agenda	1	5	2.4	1.20
8. I felt that the staff moved things along in a way that would be convenient for them	1	5	2.7	1.31
9. The staff helped me to try different positions	1	5	2.7	1.16
10. I was given time to ask questions	1	5	2.8	1.21
11. If I wanted to stop doing something, the staff allowed it	1	5	2.8	1.18
12. The staff did not pay attention to what I said	1	5	2.9	1.22
13. I could get up and walk around as much as I wanted	1	5	2.8	1.26
14. I decided on whether I would be provided with information	1	5	3.0	1.12
15. I decided on time when I would get information	1	5	3.2	1.22
16. I had control over the kind of information I received	1	5	3.3	1.19
17. Consequently, I felt the preference for the mode of birth was under my control	1	5	2.5	1.30
Perception of external control items				
18. I had control over when procedures would happen	1	5	3.2	1.12
19. I had impact on which procedures would be applied	1	5	3.1	1.19
20. I decided on whether most of procedures would be implemented	1	5	3.0	1.18
21. I had control over the decisions to be made	1	5	3.0	1.19
22. The people present in the room other than me took the control	1	5	2.7	1.09
23. I had no control on who may enter or exit the room	1	5	2.8	1.20
Perception of internal control items				
24. I was not able to control the pain I experienced	1	5	2.4	1.21
25. Pain I felt was far more than I could overcome	1	5	2.4	1.22
26. I was mentally calm	1	5	2.9	1.37
27. I could control my reactions to the pain	1	5	2.9	1.29
28. I was able to control my emotions	1	5	3.0	1.28
29. I felt that my body was doing something I could not control	1	5	2.4	1.17
30. I was under influence of negative feelings	1	5	2.8	1.28
31. I took the control by acting together with my body	1	5	2.6	1.24
32. I behaved like it was not me	1	5	2.9	1.24
33. I could control sounds I made	1	5	3.1	1.37

Abbreviation: SD, standard deviation.

There is no cut‐off point for the SCIB scale. In this study, the lowest and highest scores that can be obtained from the SCIB scale were taken into consideration and the level of support and control in birth was interpreted. The minimum score for the SCIB total score is 33 and the maximum score is 165. In this study, first, the ‘moderate level range’ was determined. For this, we subtracted the lowest score from the highest score of the scale total and divided the result by half. In this study, it was determined as the highest score of the moderate level is 132 (165:33), the lowest score is 66 (132:2) and the moderate level range is 66–132 score. After determining the moderate level range, we determined the mild and severe level ranges. According to this determination, we interpreted the mild, moderate and severe levels in this study as follows. 33–65 ‘mild’; 66–132 ‘moderate’ and ≥ 133 ‘severe’.

### 3.5 Data Analysis

The data analysis was performed on the Statistical Package for the Social Sciences 22 software. Descriptive statistics and multiple linear regression analysis were employed in the analysis of the data. Descriptive statistics included numbers, percentages, means and standard deviations. The method that is used to explain the cause–effect relationships between two or more independent variables affecting a variable with a model and to determine the effect levels of these independent variables is called multiple regression analysis. Multiple linear regression analysis was employed to investigate the impact of socio‐demographic and obstetric variables on mother's perception of control and support during birth (Tranmer and Elliot 2008). This analysis method provides insights into the linear effect of multivariate independent variables on a continuous dependent variable. In this study, when variables that yielded a statistically significant difference as a result of their comparison with the mean scores on the total mother's perceptions of control and support scale were included in the regression analysis (age, education level, number of births, mode of birth, maternal satisfaction score at birth, satisfaction with health professionals involved in birth, the health professional involved in the last birth and status of whether the health professionals fulfilled the mother's expectations). Although there was a statistically significant difference between the perceived income variable and the mean scores on the total mother's perceptions of control and support scale, this variable was not included in the regression analysis. Since the relationship between perceived income levels and the mean mother's perceptions of control and support scale score was thought to be inconsistent with the course of real life, it was not included in the multiple regression analysis. In order to perform a multiple regression analysis, certain criteria must be met. First, the multivariate normal distribution of variables was tested with kurtosis and skewness coefficients, Mahalanobis distance and Cook's distance. Also, extreme value distribution was examined. The skewness value is expected to be between −2 and +2and the kurtosis value between −10 and +10 (Collier 2020). The Mahalanobis distance of the five predictor variables should not be > 25, Cook's distance should not be > 1 and the extreme value should be < 3.3. In this study, the skewness value for regression analysis was determined to be between (0.3) and (+1.2), the kurtosis value was between (1.1) and (+2.4) and a multivariate normal distribution was assumed for the variables. In addition, the highest Mahalanobis distance was 10.08, the highest Cook's distance was 0.383 and the range of extreme values was between (1.4) and (+2.2). In the study, the multicollinearity between the variables was examined with correlation, variance inflation factor (VIF) and Durbin–Watson (D‐W) values. A correlation value of *r* < 0.75, a VIF value of < 10 and a Durbin–Watson value of D‐W = 1–3 are the desired limit values for multicollinearity (Bursal 2019). In the study, a correlation value of −0.59 to 0.47.9 (< 0.75), a maximum VIF value of 1.87 (< 10) and a D‐W value of 1.2 were found. No multicollinearity was observed between the variables. The data analysis was based on a confidence interval of 95% and a significance level of *p* < 0.05.

### 3.6 Ethical Considerations

Permission of the scale owners was obtained via e‐mail. The approval of the Scientific Research and Publication Ethics Committee of a state university was obtained (Date June 23, 2021, No: 24237859‐566). An informed consent form was provided on the first page of the online form. The consent form read the data obtained would not be used for commercial purposes and would not be shared with anyone, participation in the study would not cause any adversities and participants could quit the study at any time. Before the study was initiated, the mothers were asked if they wanted to participate in the study voluntarily in first page of the online Google form and those who responded ‘yes’ to this question started answering the questions on the questionnaire. In this study, all the mothers submitted online informed consent. All potential ethical issues associated with the use of online surveys were appropriately addressed (e.g., participants' identity and contact information were not collected and the data were stored securely online). The study was conducted in accordance with the Helsinki Declaration.

## 4 Results

### 4.1 Characteristics of the Mothers

As showed in Table 2, of the participants, 43% were aged between 26 and 30 years, 52.5% were primary school graduates, 72.7% were housewives, 90.5% had nuclear families, 53.3% lived in a city, 70% had a ‘good’ level of perceived income and 37.5% of the mothers had two births. The majority of the mothers had conceived spontaneously (89.0%) and 65.5% of the couples had wanted this pregnancy. About 52.7% of the mothers had a caesarean section and 47.3% had a vaginal birth. Twenty‐five per cent gave birth with the assistance of only a midwife, 7.5% with both a midwife and an obstetrician and 57.5% with only an obstetrician. The majority of the mothers' expectations (72.3%) from health professionals were met during birth. Maternal birth satisfaction and satisfaction with health professionals at birth were found to be above seven points on a 10‐point scale (7.47; 7.54 respectively).

**TABLE 2:** Comparison of maternal characteristics and mean sub‐dimensions and total scores of the support and control in birth questionnaire (*n* = 400).

Characteristics	*n*	%	Perception of support	Perception of external control	Perception of internal control	The support and control in birth questionnaire (total)
			Mean ± SD	Mean ± SD	Mean ± SD	Mean ± SD
Age, years						
20–25	99	24.8	50.12 ± 11.77 ^a^	19.48 ± 3.74^a^	28.14 ± 4.43	97.74 ± 16.18^a^
26–30	172	43.0	45.87 ± 7.63^b^	17.66 ± 3.15^b^	27.59 ± 4.31	91.13 ± 11.19^b^
31–35	87	21.8	45.65 ± 7.64^b^	17.69 ± 3.23^b^	27.69 ± 3.39	91.08 ± 106.1^b^
36–43	42	10.5	44.52 ± 6.96^b^	17.57 ± 2.91^b^	27.66 ± 3.88	89.76 ± 10.52^b^
*F*			6741	7550	0.390	7591
*p*			**0.000** *	**0.000** *	0.760	**0.000** *
Education level						
Primary education	210	52.5	45.01 ± 7.80^a^	17.27 ± 3.55	27.88 ± 3.99	90.59 ± 12.37^a^
High school	122	30.5	45.51 ± 8.51^b^	17.72 ± 3.25	37.36 ± 4.64	91.04 ± 11.00^b^
Undergraduate	68	17.0	47.88 ± 9.62^b^	18.14 ± 3.36	27.88 ± 3.77	94.04 ± 12.37^b^
*F*			4062	1020	0.670	3247
*p*			**0.018** **	0.362	0.512	**0.040** **
Employment status						
Yes	109	27.3	44.99 ± 7.74	17.95 ± 3.21	27.47 ± 4.15	90.42 ± 11.23
No	291	72.7	47.30 ± 9.46	18.13 ± 3.51	27.81 ± 4.17	93.25 ± 13.56
*t*			3455	0.477	0.000	4493
*p*			**0.023** **	0.642	0.467	0.052
Family type						
Nuclear	362	90.5	46.64 ± 9.05	18.08 ± 3.46	27.58 ± 4.18	92.31 ± 13.06
Extended	38	9.5	47.00 ± 9.36	18.05 ± 3.17	29.017 ± 3.78	94.13 ± 12.62
*t*			−0.232	0.061	−2115	−0.819
*p*			0.817	0.951	**0.035** **	0.413
Place of residence						
Village	33	8.3	46.96 ± 8.12	17.90 ± 3.00	26.66 ± 3.98	91.54 ± 10.68
Province	154	38.5	47.07 ± 8.98	18.00 ± 3.59	27.79 ± 4.09	92.87 ± 13.39
City	213	53.3	46.33 ± 9.30	18.17 ± 3.39	27.84 ± 4.23	32.35 ± 13.11
*F*			0.315	0.161	1167	0.164
*p*			0.730	0.852	0.312	0.849
Perception of income level						
Low	54	13.5	50.72 ± 8.71^a^	19.27 ± 3.12^a^	27.81 ± 3.94	97.81 ± 12.75^a^
High	292	70.0	46.55 ± 8.93^b^	18.03 ± 3.42^b^	27.67 ± 4.09	92.25 ± 12.68^b^
Moderate	54	13.5	43.29 ± 8.80^c^	17.16 ± 3.51^b^	27.92 ± 4.76	88.38 ± 13.54^b^
*F*			9529	5329	0.099	7479
*p*			**0.000** *	**0.005** **	0.906	**0.001** **
Number of births						
1 birth	136	34.0	45.30 ± 8.12^a^	17.75 ± 30.7	27.43 ± 4.19	90.65 ± 11.18^a^
2 births	150	37.5	45.32 ± 7.70^b^	17.89 ± 3.36	27.65 ± 3.77	90.87 ± 11.63^b^
3 births	73	18.3	45.78 ± 7.56^b^	17.95 ± 3.23	27.82 ± 3.85	92.48 ± 13.01^ab^
4+ births	41	10.3	49.16 ± 10.78^b^	18.51 ± 3.73	28.01 ± 4.42	95.69 ± 15.64^ab^
*F*			5390	1039	0.451	4326
*p*			**0.001** **	0.377	0.717	**0.005** **
Mode of conception						
Natural	356	89.0	47.15 ± 8.93	18.06 ± 3.43	27.95 ± 4.31	93.16 ± 13.33
Reproductive techniques	44	11.0	46.24 ± 9.20	18.10 ± 3.44	27.52 ± 4.02	91.87 ± 12.72
*t*			0.027	0.001	0.710	0.233
*p*			0.319	0.906	0.302	0.320
Mode of birth						
Vaginal	189	47.3	47.20 ± 9.12	18.27 ± 3.39	27.82 ± 4.12	93.30 ± 13.02
Caesarean section	211	52.7	42.38 ± 7.47	16.54 ± 3.39	26.95 ± 4.44	85.88 ± 11.07
*t*			2244	0.024	0385	1538
*p*			**0.001** **	**0.002** **	0.194	**0.000** *
Health professional involved in the last birth						
Midwife	140	35.0	48.45 ± 10.11^a^	18.31 ± 3.41	28.01 ± 4.36	94.78 ± 14.47^a^
Obstetrician (caesarean section and vaginal birth)	230	57.5	45.56 ± 8.18^b^	17.90 ± 3.44	27.60 ± 4.05	91.07 ± 11.91^b^
Midwife and obstetrician	30	7.5	46.88 ± 9.42^ab^	17.90 ± 3.44	27.60 ± 4.05	92.56 ± 12.82^ab^
*F*			4092	0.714	0.572	3244
*p*			**0.020** **	0.490	0.565	**0.044** **
Status of health professionals' fulfilling the mother's expectations during childbirth						
Yes	289	72.3	58.19 ± 9.95^a^	20.22 ± 3.91^a^	29.26 ± 4.33^a^	107.68 ± 14.06^a^
No	57	14.2	43.71 ± 6.97^b^	17.47 ± 3.17^b^	27.04 ± 4.09^b^	88.24 ± 10.40^b^
Partially	54	13.5	50.33 ± 6.85^c^	19.07 ± 3.12^b^	29.72 ± 3.25^b^	99.12 ± 9.29^c^
*F*			68,352	19,502	14,866	69,456
*p*			**0.000** *	**0.000** *	**0.000** *	**0.000** *
Maternal satisfaction score at birth	Mean ± SD					
	7.47 ± 2.01					
*r*			0.598	0.388	0.151	0.568
*p*			**0.000** *	**0.000** *	**0.002** **	**0.000** *
Satisfaction with health professionals involved in birth	Mean ± SD					
	7.54 ± 1.94					
*r*			0.597	0.304	0.179	0.554
*p*			**0.000** *	**0.000** *	**0.000** *	**0.000** *

*Note:* The evaluations of assessments of birth satisfaction are scored from 1 (lowest) to 10 (highest). ^a‐c^There is no difference between groups with the same letter for each measurement value.

Abbreviations: SD, Standard Deviation; r, Pearson correlation coefficient.

^*^ *p* < 0.01.

^**^ *p* < 0.05.

**TABLE 3:** Multiple linear regression analysis results of the mean scores on the total and sub‐dimensions of the support and control in birth questionnaire.

Variables	Perception of support				Perception of external control				Perception of internal control				The support and control in birth questionnaire total score			
	β	SE	*t*	*p*	β	SE	*t*	*p*	β	SE	*t*	*p*	β	SE	*t*	*p*
Fixed term	3.35	0.10	31.87	**0.000** *	3.47	0.18	18.56	**0.000** *	2.91	0.14	19.71	0.000	3.35	0.10	31.87	**0.000**
Age (year) (20–25)																
31–35	−0.05	0.04	−1.26	0.210	−0.21	0.07	−3.08	**0.002** **	−0.00	0.05	−0.12	0.903	−0.05	0.04	−1.26	0.210
26–30	−0.04	0.06	−0.71	0.479	−0.22	0.10	−2.07	**0.039** **	−0.02	0.07	−0.31	0.754	−0.06	0.05	−1.37	0.171
36–43	−0.06	0.05	−1.37	0.171	−0.28	0.08	−3.19	**0.002** **	0.00	0.08	0.05	0.959	−0.04	0.06	−0.71	0.479
Education (Primary education)																
High school	−0.03	0.03	−0.87	0.383	0.00	0.06	0.04	0.972	−0.02	0.048	−0.46	0.645	−0.03	0.03	−0.87	0.383
Undergraduate	0.03	0.04	0–87	0.383	0.15	0.07	1.94	0.054	0.03	0.061	0.59	0.555	0.03	0.04	0.87	0.383
Number of births (1)																
2	−0.03	0.03	−0.86	0.391	0.06	0.06	0.98	0.328	−0.03	0.05	−0.69	0.488	−0.03	0.03	−0.86	0.391
3	−0.03	0.04	−0.67	0.503	0.05	0.08	0.72	0.473	0.01	0.06	0.18	0.861	−0.03	0.04	−0.67	0.503
4+	0.04	0.05	0.77	0.441	0.17	0.10	1.64	0.102	0.00	0.08	0.11	0.915	0.04	0.05	0.77	0.441
Mode of birth (Vaginal)																
Caesarean section	0.01	0.03	0.27	0.784	0.07	0.06	1.06	0.288	−0.04	0.05	−0.84	0.401	0.01	0.03	0.27	0.784
Birth satisfaction																
Maternal satisfaction score at birth	0.06	0.01	5.83	**0.000** *	0.09	0.01	5.23	**0.000** *	0.00	0.014	0.27	0.788	0.06	0.01	−5.83	**0.000** *
Satisfaction with health professionals involved in birth	0.02	0.01	2.58	**0.010** **	0.01	0.02	0.57	0.570	0.01	0.01	1.07	0.286	0.02	0.01	2.58	**0.010** **
Health professional involved in the last birth (Midwife)																
Obstetrician	−0.01	0.03	−0.27	0.789	0.00	0.07	0.09	0.930	0.00	0.05	0.10	0.918	−0.01	0.03	−0.27	0.789
Obstetrician and midwife	−0.00	0.05	−0.00	1.000	0.04	0.10	0.47	0.639	−0.04	0.08	−0.58	0.56	−0.00	0.05	−0.00	1.000
Status of health professionals' fulfilling the mother's expectation (Yes)																
No	0.27	0.05	4.77	**0.000** *	0.19	0.07	2.34	**0.013** **	0.15	0.06	2.28	**0.023** **	0.25	0.04	5.60	**0.000** *
Partially	0.47	0.06	7.46	**0.000** *	0.20	0.08	2.50	**0.020** **	0.24	0.06	3.86	**0.000** *	0.33	0.04	6.67	**0.000** *
Model information																
*R* ^2^ Corrected *R* ^2^ *p*	0.48 0.45 0.000				0.21 0.18 0.000				0.08 0.04 0.005				0.48 0.45 0.000			

Abbreviation: SE, standard error.

^*^ *p* < 0.01.

^**^ *p* < 0.05.

It was observed that as the mothers' age, education level, and number of births increased, the level of support and control during the birth increased at a statistically significant level (*p* < 0.05). In addition, it was determined that those who gave birth vaginally, gave birth only with the help of a midwife or a midwife and an obstetrician, or were satisfied with the health professionals who assisted the birth had a higher perception of support and control during the birth (*p* < 0.05). There was a statistically significant difference between the total support and control during birth score and the mothers' birth satisfaction and satisfaction with the health professionals who assisted the birth (*p* < 0.05). No significant relationship was detected between the mothers' employment status, family type, place of residence, and mode of conception and the total support and control scale during birth score (*p* > 0.05) (Table 2).

### 4.2 Mean Scores on the Total and Sub‐Dimensions of the Support and Control in Birth Questionnaire

The mean score of the mothers on the ‘Support and Control in Birth Questionnaire’ was 92.4 ± 13.01 for the total scale, 46.6 ± 9.07 for the perception of support sub‐dimension, 18.8 ± 3.43 for the perception of external control sub‐dimension and 27.7 ± 4.16 for the perception of internal control sub‐dimension.

The minimum and maximum scores of the scale items were 1 and 5 respectively. In this study, the lowest and highest mean item scores belonged to the ‘Perception of Support at Birth’ sub‐dimension. The lowest mean score from the scale was 2.2 ± 1.01 and belonged to the item ‘The staff helped me to find the energy to continue when I wanted to give up’. The highest item score was 3.3 ± 1.19 and belonged to the item ‘I had control over the type of information I received’.

### 4.3 Examination of the Factors Affecting the Mothers' Perception of Support and Control in Birth

As seen in Table 3, multiple linear regression analysis was done with variables the mothers' age (20–25 years), education level (primary education), number of births (one), health professional involved in the last birth (midwife), and status of health professionals' fulfilling the mother's expectations (yes) on mean scores on the total and sub‐dimensions of the support and control in the birth questionnaire.

As indicated in Table 3, the analysis revealed that mothers' age, education level, number of births, mode of birth and the health professionals involved in their last birth did not show a significant impact on the total score from the perception of control and support in birth scale (*p* > 0.05). However, it was observed that the mean score on the total scale increased with higher levels of satisfaction with birth (β = 0.00, *p* = 0.000) and satisfaction with health professionals at birth (β = 0.02, *p* = 0.010). Additionally, the total score increased when mothers' expectations from health professionals at birth were met (no: β = 0.25, *p* < 0.001; partially: β = 0.33, *p* < 0.001).

Results indicated that age, education level, number of births, mode of birth and the health professional involved in the mother's last birth did not have a significant effect on sub‐dimensions, either (*p* > 0.05). The perception of support increased as the level of maternal satisfaction with birth (β = 0.06, *p* < 0.001) and health professionals at birth increased (β = 0.02, *p* = 0.010) and as their expectations from the health professionals at birth were met (no: β = 0.27, *p* < 0.001; partially: β = 0.47, *p* < 0.001).

Maternal education level, number of births, mode of birth, health professional involved in birth and birth satisfaction were not significantly effective in the perception of external control at birth sub‐dimension (*p* > 0.05). However, the perception of external control decreased with progressing maternal age (*p* < 0.05) and the level of fulfilment of expectations from maternity health professionals (no: β = 0.19, *p* = 0.013; partially: β = 0.20, *p* = 0.020) (Table 3).

Maternal age, education level, number of births, mode of birth, health professionals who assisted the birth and satisfaction with birth did not show a significant effect on the perception of internal control at birth sub‐dimension (*p* > 0.05). Nevertheless, it was observed that the perception of internal control increased as the level of fulfilment of mothers' expectations from health professionals increased (no: β = 0.15, *p* = 0.023; partially: β = 0.24, *p* < 0.001) (Table 3).

## 5 Discussion

This study aimed to assess the level of perception of control and support at birth and investigate the factors influencing it. The mean score of the mothers in this study was found to be 92.4 ± 13.01 on the total SCIB score (minimum: 39, maximum: 138). Considering the range of scale scores from 33 to 165, it can be inferred that the maternal perceptions of control and support at birth in this study fell within a moderate level. As explained in the methods section of this study (see 3.4 Instrument), if the total SCIB score is ‘66–132 range’, this was interpreted as moderate level. In comparable studies conducted with the same scale, the mean score on the total SCIB ranged from 99.04 ± 17.3 to 120 (Aynaci 2020; Demirel, Kaya, and Evcili 2022; Cevik et al. 2023; Shahveisi, Nourizadeh, and Mehrabi 2023). The total score of the SCIB in studies by Demirel, Kaya, and Evcili (2022) and Cevik et al. (2023) was similar to our results, while Aynaci (2020) and Shahveisi, Nourizadeh, and Mehrabi (2023) reported higher scores than our values. Variations in study findings may be attributed to factors, such as the socio‐demographic and cultural characteristics of mothers, the quality of services provided by health personnel during birth, the facilities of the birth unit, practices employed during birth and the quality of antenatal care.

The SCIB comprises three sub‐dimensions, namely perception of support, perception of external control and perception of internal control. In our study, the mean scores from these sub‐dimensions were found to be 46.6 ± 9.07, 18.8 ± 3.43 and 27.7 ± 4.16 respectively. A comparison with a study involving primiparous mothers in Iran revealed that while the perception of internal control score was similar to that of our study (28.5 ± 2.73), the perception of external control score was higher (31.2 ± 4.54) and the perception of support score was lower (23.3 ± 3.76) (Shahveisi, Nourizadeh, and Mehrabi 2023). In a study conducted in Gambia with a sample consisting of 74% multiparous mothers, it was found that mothers' internal control was higher than their external control (Colley et al. 2018). Additionally, in a study involving Syrian adolescent immigrant mothers, it was determined that maternal perception of external control was higher than internal control (Cevik et al. 2023). It is necessary to consider factors, such as the socio‐demographic, obstetric and cultural characteristics of mothers, when interpreting the perception of control and support scores. In some cultures, there is a belief that birth pain is normal and that there is no need for external control or medical support to cope with it. In a study, it was reported that some birth unit employees perceived birth pain as natural and normal, suggesting that painkillers did not need to be administered unless the pain was severe (Buback et al. 2022). Therefore, it is crucial to assess the perception of support and control at birth from a cultural perspective.

In our study, it was determined that the lowest and highest scores from SCIB were obtained from the ‘perception of support’ sub‐dimension. The item ‘The staff helped me to find the energy to continue when I wanted to give up’ on the perception of support sub‐dimension yielded the lowest score. This finding suggested that the support and supportive care received from the maternal health professionals was inadequate. In some studies, mothers stated that they had not received enough physical and emotional support from midwives during birth and therefore had a negative experience (Aktaş and Aydın 2019; Buback et al. 2022, Toker & Aktaş, 2021).

Inadequate support from health professionals during birth reduces the ability to cope with birth pain and birth control, increases the need for medical interventions and reduces birth satisfaction (Viirman et al. 2023; Olza et al. 2020). In our study, the highest item score was obtained from the ‘I had control over the type of information I received’ of the social support sub‐dimension. This finding showed that mothers had control over the issues they needed to receive ‘information support’ during birth. Mothers' knowledge of what information they will need during birth and receiving care accordingly increases maternal satisfaction and the quality of care during birth (Aktaş and Pasinlioğlu 2021). In a study by Turan, Suveren, and Vural (2022), some mothers stated that the information support provided by midwives regarding the issues they needed during birth increased ‘satisfaction with the midwife and birth’.

In our study, maternal perceptions of support were higher than perceptions of internal and external control. While this finding is consistent with some studies (Demirel, Kaya, and Evcili 2022; Cevik et al. 2023), it contradicts some others (Aynaci 2020; Shahveisi, Nourizadeh, and Mehrabi 2023). In our study, 34% of the mothers were primiparous and 52.7% had a caesarean section. Whether those with caesarean sections had emergency or planned procedures was not investigated in the study. Those undergoing emergency caesarean sections received more support from health professionals. The physical and emotional support provided by healthcare professionals during birth enhances maternal control and birth satisfaction (Mortazavi and Mehrabadi 2024; Aktaş and Pasinlioğlu 2021). In a randomised controlled study, it was found that birth dance, applied to pregnant women by a health professional in the first stage of birth, facilitated coping with birth pain, increased birth control, provided emotional support and enhanced birth satisfaction (Kaloğlu, Binici, and Aktaş 2023). It was also suggested in the literature that women trained by midwives and obstetricians in non‐pharmacological practices during birth had higher perceptions of control and support at birth (Shahveisi, Nourizadeh, and Mehrabi 2023). Striebich and Ayerle 2020 highlighted that pregnant women expressed a desire to understand how they could contribute to a physiological birth, had concerns about losing control during birth and sought information to maintain control.

In our study, fulfilment of maternal expectations from health professionals during birth had a positive impact on mothers' perceptions of support, internal control and external control. This suggests that mothers whose expectations are fulfilled during birth may experience high birth satisfaction through high support and control. In Turkish society, mothers typically expect respect, empathetic communication, a smiling face, guidance (e.g., in pain management and pushing), support, comfort and a compassionate approach from midwives during birth (Aktaş and Pasinlioğlu 2021; Turan, Suveren, and Vural 2022; Aktaş and Küçük Alemdar 2024). A systematic review of 51 studies indicated that the support provided by companions, health professionals and partners during birth contributed to emotional, informational and physical support, including activities such as giving a massage and holding hands, instilling confidence, enhancing support and control during birth and fostering a positive birth experience (Bohren et al. 2019). The support offered by health professionals promotes relaxation in mothers and contributes to increased satisfaction with the birth process. Aktaş and Pasinlioğlu (2021) found that empathy training for midwives in the birth room led to increased relaxation, active participation in decision making and information sharing, meeting expectations and overall maternal satisfaction with birth experience.

Our results indicated that an increase in mothers' satisfaction with birth had a positive impact on the overall score of control and support during birth and the perception of support sub‐dimension score. This finding suggested that mothers whose expectations were met by healthcare professionals experienced a high perception of control and support during birth. Of the mothers in our study, 35% had a vaginal birth led only by a midwife. Current literature suggests that expert midwifery care during pregnancy and birth not only provides social support for women but also instils confidence, enhances the sense of control during birth (such as managing birth pain and bodily aspects of birth) and contributes to a positive birth experience. (Moloney and Gair 2015; Bäckström et al. 2016). The supportive care provided by midwives during birth has been associated with an increase in women's external control (Çankaya and Can 2021). In one study, the majority of primiparous mothers who were satisfied with the midwife‐assisted vaginal birth expressed a preference for a vaginal birth for their subsequent birth (Aktaş and Pasinlioğlu 2017). Shahveisi, Nourizadeh, and Mehrabi (2023) investigated the impact of joint decision making on the choice of analgesia during birth in primiparous mothers. The study revealed that mothers involved in joint decision making had higher birth support and internal and external control scores along with positive birth experience and increased satisfaction with birth. The participation of mothers in decision making during birth not only provides support but also enhances their sense of control and satisfaction with the birth process.

In this study, the method of birth did not have a significant effect on mothers' perceived support and control during birth. Nearly half of the participants in our study (47.3%) had a vaginal birth. While some studies indicate that the mode of birth does not influence maternal perceptions of control and support (Demirel, Kaya, and Evcili 2022), some others suggest that mothers who deliver vaginally tend to have a high sense of support and control compared to those undergoing a caesarean section (Colley et al. 2018; Aynaci 2020). It is important to note that differences in study findings may have stemmed from various factors, such as cultural differences, birth centre facilities, quality of care and maternal expectations.

Several variables, including socio‐demographic, cultural, obstetric factors and antenatal and intrapartum care services, may impact maternal perceptions of control and support during birth (Shahveisi, Nourizadeh, and Mehrabi 2023; Buback et al. 2022; Olza et al. 2020). Our study revealed that an increase in maternal age and satisfaction scores correlated with a decrease in perceived external control during birth. Conversely, factors like educational level, employment status and the number of previous births did not yield statistically significant effects on the total and sub‐dimension scores (support, internal and external control) of the perception of control and support in birth scale. There are both corroborating and divergent findings concerning our study in the literature. Colley et al. (2018) found that mothers who were aged ≥ 25 and had vaginal birth exhibited a higher perception of internal control. Similarly, Green and Baston (2003) established a robust correlation between the number of pregnancies and the perception of control, highlighting that multiparous mothers tended to have a greater sense of control than primiparous mothers. Notably, in our study, a majority of mothers were multiparous and their perception of internal control surpassed that of external control. The concept of internal control perception encompasses the ability to manage the body during birth, cope with birth pain and navigate emotions, among other factors. It is plausible that the high perception of internal control among multiparous mothers in our study can be attributed to their prior birth experiences, potentially influencing their ability to manage the various aspects of birth.

### 5.1 Implications for Midwifery and Nursing Practice

Perception of control and support during birth is closely related to quality of care, birth satisfaction and birth experience. Low levels of perception of control and support can lead to negative outcomes, such as inability to cope with labour pain, increased need for medical interventions and requests for caesarean section, prolonged labour and negative and traumatic birth experiences. Therefore, midwives and nurses need to assess women's perceptions of control and support during birth starting from the antenatal period to identify and prevent possible risks early.

Midwives and nurses, who are the primary providers of obstetric care, should provide antenatal and intrapartum care that helps determine and meet the psychosocial needs of women and families to increase the perception of support and control during birth and should increase their coping skills with labour pain and birth self‐efficacy through education and counselling services. During the birth process, midwives should provide a birth environment that protects birth physiology and increases maternal satisfaction during birth (such as the width of the birth space/room for freedom of movement during birth, the heat and light source of the room, the presence of noise in the environment, the comfort of companions and the harmony of the birth team). It should be kept in mind that birth companions (such as spouses and friends) can affect the mother's perception of social support and that the woman and her family should be provided with care with a holistic approach. Midwives and nurses should conduct more studies on this subject with mixed designs and reconsider their care protocols in the obstetric field in line with these studies.

### 5.2 Strengths and Limitations of the Study

The strength of the study was that it covered mothers from every region of Türkiye. The study has several limitations. One of these limitations was that mothers gave birth in the maternity units of different hospitals (with 98% of births in Türkiye performed in hospitals). Mothers' perceptions of control and support during birth may vary depending on the maternity units and the health professionals working in these units. Another limitation was that only mothers with smartphones participated in the study.

## 6 Conclusion

The results of this study indicated that maternal perceptions of control and support during birth fell within a ‘moderate’ level, considering the lowest and highest scores on the Support and Control in Birth Scale. Notably, an increase in satisfaction with the birth experience and healthcare professionals, including midwives and obstetricians, positively influenced maternal perceptions of control and support during birth. In contrast, certain socio‐demographic and obstetric characteristics did not demonstrate a significant impact. It is recommended that future research focus on analysing maternal perceptions of control and support, considering the factors influencing these perceptions, through mixed method studies. Also, comparative studies in larger populations are recommended.

## Author Contributions

**Tuğba Yazici Topçu**, **Ruveyde Aydin** and **Songül Aktaş:** study conception, study design, drafting the manuscript, critical revisions for intellectual content and supervision; **Tuğba Yazici Topçu:** data collection; **Ruveyde Aydin** and **Songül Aktaş:** data analysis.

## Conflicts of Interest

The authors declare no conflicts of interest.

## Supporting information


Data S1.

## Data Availability

The data that support the findings of this study are available on request from the corresponding author on reasonable request.
